# Breaking the Fatigue Cycle: Investigating the Effect of Work-Rest Schedules on Muscle Fatigue in Material Handling Jobs

**DOI:** 10.3390/s23249670

**Published:** 2023-12-07

**Authors:** Karla Beltran Martinez, Milad Nazarahari, Hossein Rouhani

**Affiliations:** Department of Mechanical Engineering, University of Alberta, Donadeo Innovation Centre for Engineering, Edmonton, AB T6G 1H9, Canada; beltranm@ualberta.ca (K.B.M.); nazaraha@ualberta.ca (M.N.)

**Keywords:** electromyography, musculoskeletal disorders, wearable inertial measurement units, muscle fatigue, work–rest schedule

## Abstract

Muscle fatigue has proven to be a main factor in developing work-related musculoskeletal disorders. Taking small breaks or performing stretching routines during a work shift might reduce workers’ fatigue. Therefore, our objective was to explore how breaks and/or a stretching routine during a work shift could impact muscle fatigue and body kinematics that might subsequently impact the risk of work-related musculoskeletal disorder (WMSD) risk during material handling jobs. We investigated muscle fatigue during a repetitive task performed without breaks, with breaks, and with a stretching routine during breaks. Muscle fatigue was detected using muscle activity (electromyography) and a validated kinematic score measured by wearable sensors. We observed a significant reduction in muscle fatigue between the different work–rest schedules (*p* < 0.01). Also, no significant difference was observed between the productivity of the three schedules. Based on these objective kinematic assessments, we concluded that taking small breaks during a work shift can significantly reduce muscle fatigue and potentially reduce its consequent risk of work-related musculoskeletal disorders without negatively affecting productivity.

## 1. Introduction

Material handling, i.e., transporting material from one point to another manually, is still essential in many industries [1]. Performing improper lifting or overexertion during material handling jobs is one of the main causes of musculoskeletal disorders (MSDs) [2]. MSDs are injuries that can affect the muscles, nerves, tendons, joints, cartilage, and/or spinal disks [3]. Around 20% of people worldwide suffer from some type of MSD [4], which turns MSDs into a global health issue. When an MSD occurs or is significantly worsened in the work environment, it is known as a work-related musculoskeletal disorder (WMSD).

In the present day, engineers continue to bear a moral, legal, and ethical obligation to safeguard the well-being of the public, both in their professional conduct and in the development of processes, labor, and work environments [5]. Exposure to task repetition, forceful exertion, and sustained awkward postures can cause fatigue and eventually lead to an MSD [6,7]. Thus, to reduce the risk of fatigue-induced WMSDs, it is important to include a recovery break after feeling fatigued, to recover the muscles from fatigue [7]. While each person feels fatigued at a different pace, analyzing factors such as fitness habits, general health, and work practices can help us detect fatigue in the individual [7,8]. From a physiological perspective, muscle fatigue occurs when the maximal force capacity starts to decline [9]; this forces an increase in motor units, which shows as an increase in the electromyography (EMG) amplitude [10].

One of the most popular methods to reduce work-related fatigue is job rotation. However, job rotation has several limitations, such as not being applicable to workers with medical restrictions (e.g., physical impairment) or of decreasing product quality [11]. Another method for fatigue prevention is the implementation of engineering controls, such as redesigning workstations, which has proven to be an effective technique for reducing WMSD. Nevertheless, this approach involves a high level of investment and a complicated redesign process that industrial companies are not always willing to perform [12]. Last, work–rest schedules have been proposed to reduce work-related fatigue [13]; for instance, having a 30 s break every 40 min of work [14] or an additional 5 min break for every hour without a break [15]. Previous studies have suggested many different work–rest schedules to prevent fatigue during a work shift and have validated them through workers’ interviews [16,17,18,19], or energy consumption [20,21]; however, the impact of fatigue, breaks, stretch on muscles activities, and body kinematics should be further studied.

Performing stretching exercises has also been proposed to reduce fatigue-induced WMSDs. For instance, an observational rating scale has shown that a stretching routine can reduce the risk of fatigue for certain activities [22]. Also, muscle fatigue detection during gas cylinder handling tasks was studied via electromyography (EMG) recordings to demonstrate the benefits of including a stretching routine at the beginning of the task execution [23]. However, this study obtained inconclusive results as (1) the tasks were not long enough to induce muscle fatigue, and (2) they did not provide enough rest to allow muscle recovery between trials with and without a stretching routine [23].

Therefore, to complement the existing literature, we aimed at objectively investigating whether using breaks and/or a stretching routine during a work shift can reduce muscle fatigue during material handling jobs. Despite previous efforts to study this research question [24,25,26], there is still a need for an objective tool to prove if specific work–rest schedules can reduce muscle fatigue. In our last study [27], we introduced a postural phenomenon that occurs during fatigue measured with IMU sensors. In this paper, we want to (1) assess the ability of our system to detect muscle fatigue via inertial measurement units (IMUs) during a standard material handling job with three different work–rest schedules and validate it with an EMG sensor, and (2) compare the muscle fatigue among these three schedules to quantify the impact of each schedule on muscle fatigue and potentially the risk of fatigue-induced WMSDs.

## 2. Materials and Methods

### 2.1. Experimental Study

**Figure 1:**
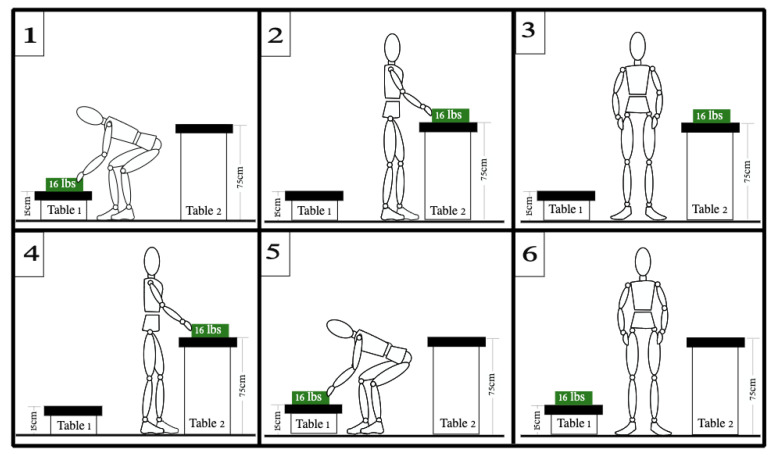
Description of the experimental setup: (**1**) Participant grasps a 16 lb. load from a Table 15 cm in height (Table 1); (**2**) Participant turns left and places the load on a different Table 75 cm in height (Table 2); (**3**) Participant goes to the neutral position; (**4**) Participant takes the load back from Table 2; (**5**) Participant turns right and moves the load to Table 1; and (**6**) Participant goes back to the neutral position. This figure is adopted and modified from our previous article [27]. Permission is obtained from the publisher.

We designed an experimental setup and procedure, described in our previous work [27], to physically exhaust participants during a standard material handling job while measuring their muscle fatigue levels with two different tools: EMG recordings, and a kinematic score (K-score) measured using wearable IMUs. Nine able-bodied participants (all male, age: 24 ± 2, body mass: 71 ± 9 kg, body height: 177 ± 2 cm) performed a material handling job. Each participant moved a 16 lb. box (7.2 kg) from a Table 15 cm in height to a second Table 75 cm in height and back (see Figure 1 for more details on the experimental setup). Participants were asked to rate their level of tiredness every 2 min on a 0 to 10 scale using the RPE CR-10 scale [28], and when they reached a score of 9 or 10, the trial was stopped. Participants performed the task at their own preferred pace. The load used in this experiment was calculated using the National Institute of Occupational Safety and Health (NIOSH) lifting equation [29], based on the tables’ heights and the quality of the load grasp. The experiment was approved by the local ethics committee at the University of Alberta (Pro00089234). The participants provided written consent prior to the experiments.

### 2.2. Work–Rest Schedules

The experiment above was repeated in three different work–rest schedules: Schedule 1 was conducted without any rest and was considered the control schedule. Based on Schedule 1, we calculated the (1) duration for each participant to perform the task before reaching fatigue, and (2) how much the muscles were fatigued when no breaks were included.

**Figure 2:**
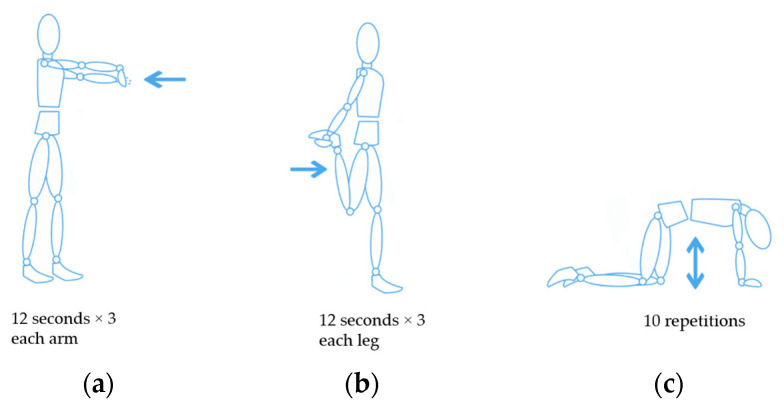
Stretching routine implemented in Schedule 3: (**a**) Participants straightened their arms in front of them and pulled their fingers back with the opposing hand; (**b**) Participants flexed their legs backward and pulled their feet with their arm; (**c**) Participants started with hands and knees on the floor and moved their spinal column to a round position toward the ceiling, with their chin toward their chest, then moved from this position to the opposite (i.e., approached their abdomen near the floor and head backward).

For Schedules 2 and 3, a 1 min microbreak was given to participants after each one-third of the trial. For example, if a participant felt fatigued after 30 min during Schedule 1, the breaks for Schedules 2 and 3 were given every 10 min. For Schedule 2, the participant sat still during the microbreak. For Schedule 3, a stretching routine, composed of the three movements shown in Figure 2, was performed. This stretching routine was chosen based on the muscles that showed most fatigue during a previous study [27], and the most common stretching exercises in repetitive work according to [30].

**Figure 3:**
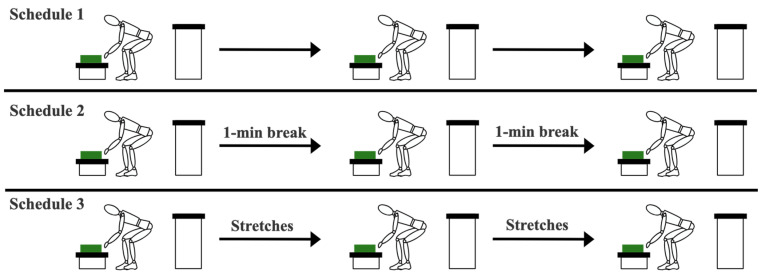
Difference between the three schedules: Schedule 1: perform the task continuously; Schedule 2: have a 1 min break after every one-third of the trial; and Schedule 3: perform the stretching routine shown in Figure 2 after every one-third of the trial. The green boxes represent the load being carried.

The trials were always performed in the order of Schedule 1, Schedule 2, and Schedule 3, and are further explained in Figure 3. Also, we considered a waiting period of at least two days between the three experimental trials of each participant to allow for muscle fatigue recovery [31].

### 2.3. Fatigue Analysis Based on Muscle Activity Recording

**Figure 4:**
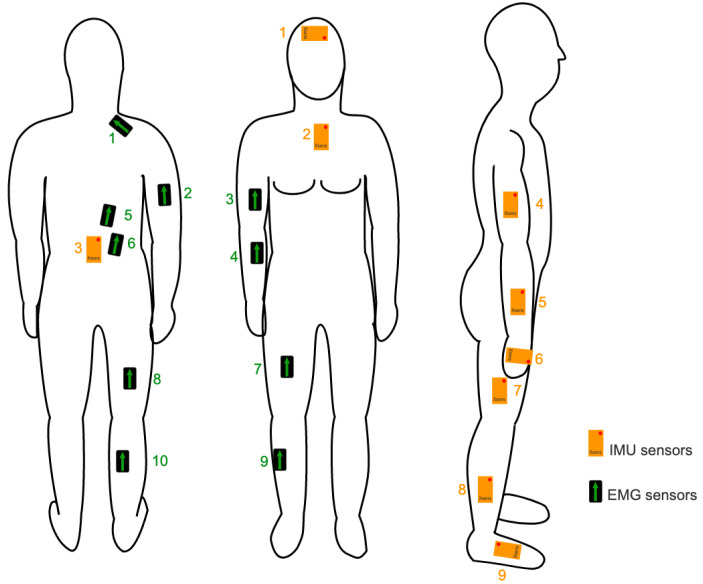
Location of IMU (orange boxes) sensors: (1) head, (2) chest, (3) pelvis, (4) upper arm, (5) forearm, (6) hand, (7) thigh, (8) shank, and (9) foot; and location of EMG (black boxes) sensors: (1) trapezius, (2) triceps, (3) biceps, (4) carpi radialis, (5) and (6) erector spinae, (7) rectus femoris, (8) bicep femoris, (9) tibialis anterior, and (10) lateral gastrocnemius.

Participants’ muscle activity was recorded using wearable EMG sensors (Trigno, Delsys, Natick, MA, USA) at 1200 Hz to detect muscle fatigue during the experiments. EMG sensors were attached to the biceps, flexor carpi radialis, rectus femoris, tibialis anterior, biceps femoris, lateral gastrocnemius, erector spinae, triceps, and trapezius, as shown in Figure 4. Each muscle provided a one-dimensional data representation. The EMG electrode location was shaved and cleaned with alcohol swabs before attaching the sensors, to enhance the quality of the EMG recordings. As this study focused solely on individual comparisons, a maximum voluntary contraction (MVC) procedure was not conducted.

The EMG recording time series were filtered with a 4th-order band-pass Butterworth filter, with cutoff frequencies of 10 Hz and 500 Hz; subsequently, the time series was smoothed and rectified [10,32]. The root-mean-square (RMS) amplitude of the time series during each repetition (one repetition: moving the box from one table to another and back) was considered as the EMG amplitude and was used for muscle fatigue detection following the recommendations in [10,33]. We detected muscle fatigue when the mean EMG amplitude increased more than 5% between the first and last 10% of a trial [27].

**Figure 5:**
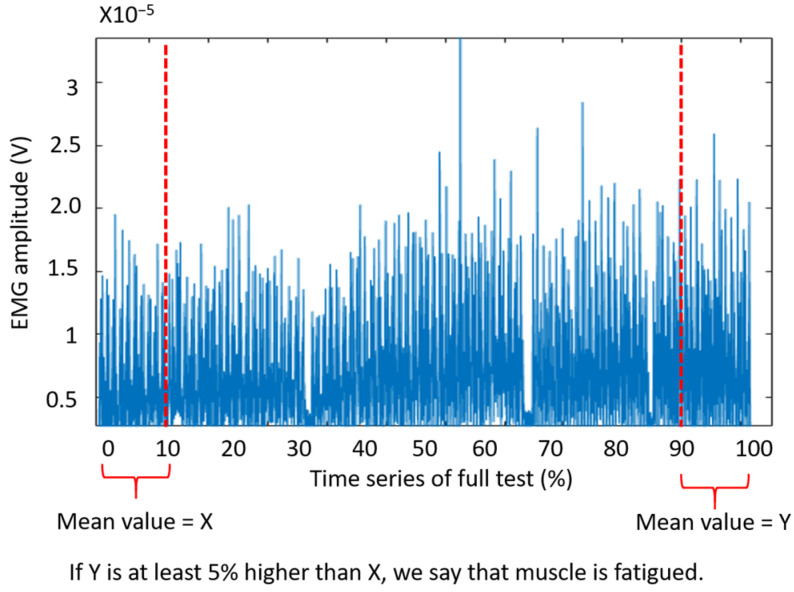
RMS time series of EMG amplitude recorded for carpis radialis of a representative participant during a full trial of Schedule 1. X and Y represent the mean value of the EMG amplitude during the first and last 10% of the trial, respectively. Fatigue was detected if Y > 1.05 × X.

For instance, Figure 5 shows the flexor carpis radialis activity during a representative trial and how to detect fatigue based on a change in EMG amplitude. Once we identified the fatigued muscles (e.g., muscles A and B) and the percentage of their EMG amplitude increase (e.g., X% and Y%, respectively), we added these percentages for the fatigued muscles and obtained an accumulative fatigue measurement (i.e., the accumulative fatigue score = (X + Y)). For example, if a participant showed 26% and 18% increase in EMG amplitude of the bicep femoris and erector spinae, respectively, and did not show fatigue in any other muscle, then the accumulative fatigue score was calculated as 44 = 26 + 18. We observed that a change of EMG amplitude for less than 5% can occur due to various reasons, and might not be a true indicator of muscle fatigue. Therefore, the inclusion of muscles with an EMG amplitude change of less than 5% in the fatigue score could make the fatigue score sensitive to various sources of error. Thus, we did not include non-fatigued muscles in the fatigue score; see [27] for more details.

### 2.4. Fatigue Analysis Based on Body Posture and Kinematics Measurement

In addition to fatigue detection with EMG, we calculated a kinematic score (i.e., K-score) proposed in our previous work [27] to identify fatigue. To this end, we measured joint angles (trunk, knee, shoulder, and elbow) using nine IMUs (Xsens, Movella, Henderson, NV, USA) attached to the forehead, sternum, sacrum, upper arm, forearm, hand, thigh, shank, and foot, as shown in Figure 4. Each IMU measured the orientation of the segment it was attached to with a sampling frequency of 100 Hz. Both the EMG and IMU signals were synchronized to each other. The orientation of each IMU was obtained using the Xsens proprietary sensor fusion algorithm, validated for similar manual handling tasks [34,35].

Then, to obtain the body segments’ orientation in the anatomical frames (required for anatomically meaningful joint angle representation), we used a sensor-to-segment frame transformation through a functional calibration procedure [36,37]. The functional calibration procedure was performed at the beginning of each experiment and included five seconds of standing still, followed by flexion–extension motions of the leg and arm in the sagittal plane. Then, the 3D joint angles were calculated according to the joint coordinate system [38].

**Figure 6:**
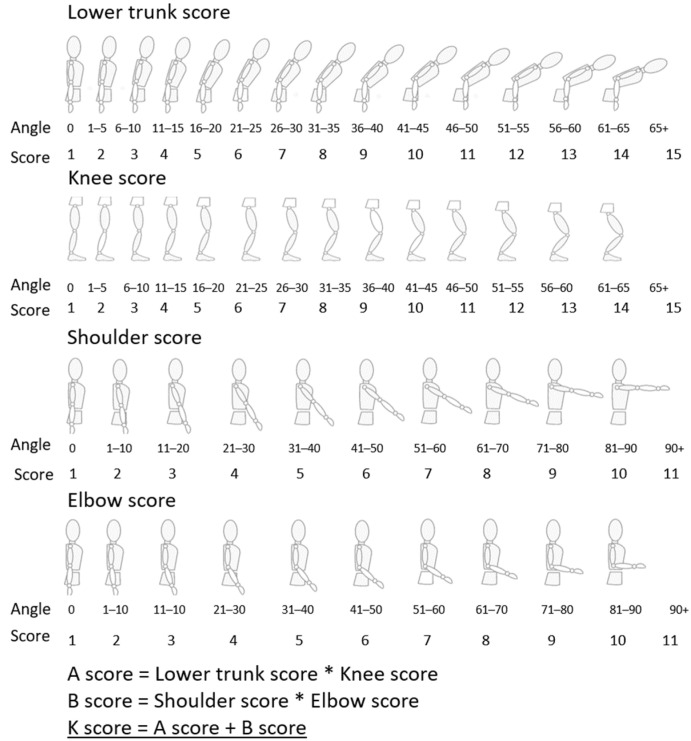
Step-by-step worksheet to measure the proposed K-score using the joint angles. This figure was modified from our previous article [27]. Permission was obtained from the publisher.

To cancel the drift in the segment orientations estimated by the IMUs, the participants were asked to stand still in the N-pose for 3 s every 5 min, and we assumed that all the joint angles were equal to zero during this period. This brief pause was also used to determine if the trial should continue or stop, based on the participants’ levels of fatigue. Once the joint angles were calculated, the K-score was obtained for each time instant, as described in Figure 6; more information can be found in [27]. We calculated the RMS of the K-score during each repetition, and then these RMS values for the first and last 10% of each trial were compared to detect fatigue, similar to the fatigue detection based on the EMG recordings.

### 2.5. Data Analysis

In addition to fatigue analysis using muscle activity and body kinematics measurements, the productivity of each schedule was measured by counting the number of repetitions during the schedule’s implementation. The calculated parameters, based on the EMG and IMU recordings, did not show a normal distribution. Therefore, to test for significant differences in the detected muscle fatigues (characterized by the EMG and IMU recordings), in different work–rest schedules, we employed the Wilcoxon signed rank test with a significance level = 5% between each pair of the three schedules (Schedule 1 against Schedule 2, Schedule 1 against Schedule 3, and Schedule 2 against Schedule 3).

## 3. Results

**Figure 7:**
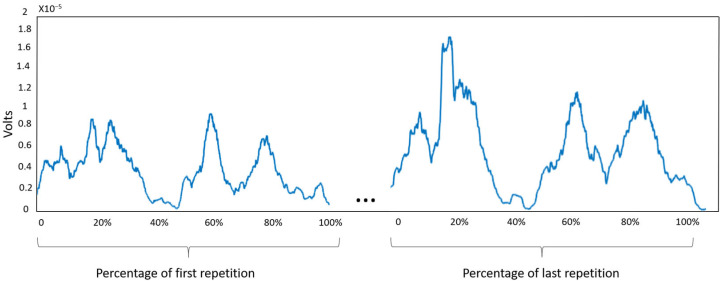
An example of EMG signal of erector spinae iliocostalis of a participant during the first and last repetition of Schedule 1.

The accumulative fatigue measured based on EMG recordings were [111.7, 134.3, 168.0]% ([25th percentile, 50th percentile, 75th percentile] among all participants) for Schedule 1, [29.5, 52.8, 66.6]% for Schedule 2, and [33.6, 57.6, 107.6]% for Schedule 3 (Table 1). We reported the data in these percentiles instead of the mean value and standard deviation, because the data distribution was not normal. Moreover, Figure 7 illustrates an example of the variations in EMG amplitude throughout the trial, specifically highlighting the fatigue-induced changes in the erector spinae iliocostalis muscle of a participant during the initial and concluding repetitions of Schedule 1.

**Figure 8:**
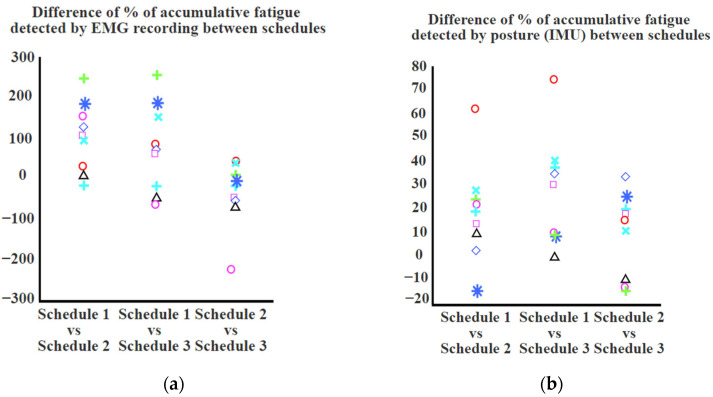
Difference of muscle fatigue detected between Schedule 1 and Schedule 2 and between Schedule 1 and Schedule 3 for all participants measured with (**a**) EMG amplitude and (**b**) Body posture. The results for each participant are shown with a unique symbol and/or color.

The accumulative fatigue measured with the K-score (body posture) were [30.7, 40.8, 43.8]%, [19.3, 25.6, 26.9]%, and [4.9, 11.8, 19.2]% for Schedules 1, Schedule 2, and Schedule 3, respectively. The Wilcoxon signed rank test showed a significant reduction (*p* < 0.05) in muscle fatigue between Schedule 1 and Schedule 2 and between Schedule 1 and Schedule 3 measured with both body posture and EMG recordings (Figure 8). Neither the EMG nor the body posture showed a significant difference in muscle fatigue between Schedule 2 and Schedule 3. The full time for each participant to complete the trial varied between 15 and 60 min, with a mean value of 35.5 min. The productivity of each schedule was [198, 235, 384], [209, 279, 345], and [187, 245, 441] for Schedules 1, Schedule 2, and Schedule 3, respectively (Table 2). The Wilcoxon signed rank test showed no significant difference between the productivity of the three schedules.

## 4. Discussion

An efficient work–rest schedule is an easy and inexpensive method to reduce muscle fatigue and subsequently the risk of WMSDs [24]. Recent studies presented work–rest schedules that included stretch routines and showed their potential to reduce muscle fatigue [30]. However, these studies used the participants’ feedback to detect fatigue, which is inherently subjective and has limited sensitivity. Also, it has been shown that, by performing stretches in the workplace, employees can improve their flexibility and overall sense of well-being [39]. However, this study did not confirm that improving flexibility can reduce the risk of WMSDs, since the experiment lacked a control schedule and the tasks were not long enough to observe any meaningful changes in muscle fatigue.

To address these limitations, our study presents a systematic approach toward (1) the objective measurement of muscle fatigue in long-duration trials using wearable EMG sensors and IMUs, and (2) assessing the effect of work–rest schedules on reducing muscle fatigue during material handling tasks. We compared three different work–rest schedules: Schedule 1: task execution without any breaks, Schedule 2: task execution with two 1 min microbreaks, and Schedule 3: task execution with two microbreaks, which included a stretching routine.

Furthermore, studies like [40,41] have developed objective models to propose a work–rest schedule, or rest allowance based on the energy expenditure rates during the working and resting periods, while these values were taken from the oxygen intake of the worker. Even though this model was objective, it did not consider the differences among participants’ physiological characteristics, and could only be used for tasks that require sufficient intensity to disturb the oxygen intake [21]. Our instrumented fatigue detection method complements this work with the possibility of personalizing the time intervals in which the breaks should be given to an individual, toward recommending an individual-specific work–rest schedule to minimize muscle fatigue while preserving productivity. Yet, this application should be further investigated in the future.

Muscle activity monitoring with surface EMG sensors is the most common technique for measuring fatigue [1,42]. However, this technique can be difficult to implement during long-duration material handling tasks and in industrial environments where movement artifacts and sweating can decrease the signal-to-noise ratio in EMG recordings. Furthermore, when dealing with dynamic tasks, the analysis of EMG signals is limited to the signal amplitude alone, which lacks the accuracy provided by analyzing both amplitude and frequency, as is possible in static tasks [43]. Therefore, we validated the use of wearable IMUs to measure muscle fatigue during different work–rest schedules, which could be a reliable substitution for EMG sensors.

Table 1 shows that 8 and 6 (out of 9) participants experienced a reduction in their muscle fatigue detected with EMG during Schedule 2 and Schedule 3, respectively, compared to Schedule 1. The maximum reduction in muscle fatigue was 93% during Schedule 2 compared to Schedule 1 for participant 6. However, participants 7, 8, and 9 showed an increase in muscle fatigue during Schedule 2 and Schedule 3 compared to Schedule 1. Table 1 also shows that the maximum reduction in muscle fatigue measured with the body posture (K-score) was 93% during Schedule 3 compared to Schedule 1 for participant 4. There was also an unexpected increase in muscle fatigue (measured with the K-score) of participant 3 during Schedule 2 compared to Schedule 1. This unexpected increase could be due to the large gap between data collection sessions (up to two months instead of two days) for the three schedules for participants 7, 8, and 9, due to restrictions caused by the COVID-19 pandemic. The change in the participants’ lifestyle during this period (mostly reduction in regular physical activity due to quarantine) could have affected their functional capacity and, consequently, fatigability.

Regardless of the abovementioned inconsistency of the observed muscle fatigue in Schedules 2 and 3 among participants, the Wilcoxon signed rank test showed a significant difference (*p* < 0.05) in muscle fatigue between Schedule 1 and Schedule 2 and between Schedule 1 and Schedule 3 (based on both the K-score measured by IMUs and the EMG measurements). These differences reveal the effectiveness of work–rest schedules on muscle fatigue reduction. Furthermore, there was no significant difference between muscle fatigue (based on both body posture measured by the IMUs and EMG measurements) in Schedules 2 and 3. Therefore, we did not observe evidence for the effectiveness of stretching routines during microbreaks on muscle fatigue reduction. Overall, the results of our experimental study indicate that even a small break can be considered a preventive measure to reduce fatigue and, in turn, the risk of MSDs, without affecting the productivity of the work. Also, we observed similar trends in muscle fatigue change between Schedules 1, 2, and 3 based on both K-score measured by IMUs and EMG measurements. This could indicate that wearable IMUs can be a reliable tool to measure muscle fatigue based on body posture changes in repetitive tasks and can substitute EMG sensors.

The current study had a number of limitations. First, some experiments were interrupted due to the COVID-19 pandemic, causing a few weeks gap between experiments for Schedule 2 and/or Schedule 3. The participants’ change in physical activity level and lifestyle might have impacted the fatigability of muscles. Yet, the results of our non-parametric statistical tests were little impacted by the inconsistency of the measured outcome of a few participants compared to others. Second, the movement pace during the trials was not controlled. Third, the daily level of the physical activity of each participant was not recorded, and neither was their general health nor regular work habits. Additionally, all the participants had a similar age. Therefore, our study participants might not be representative of our target worker population with manual material handling tasks. All of these factors can impact the fatigability of muscles. Fourth, the definition of fatigue given on this study has not been used before and needs to be studied further. Fifth, the order of the Schedules was not controlled, and could have a learning effect for Schedules 2 and 3. Last, the number of participants and their work experience for this study is limited and should be extended in the future. Despite these limitations, our observations showed the potential of determining the impact of the work–rest schedule on muscle fatigue based on measurements of EMG sensors and IMUs. Investigating the impact of break (with or without stretching) duration and frequency on muscle fatigue, however, requires experimental studies (1) during a multi-hour shift in an industrial environment to characterize these impacts during a full work shift, and (2) with a larger population and including female participants for making general conclusions.

## 5. Conclusions

We investigated the impact of microbreaks and stretching routines on muscle fatigue reduction and showed that a work–rest schedule including 1 min microbreaks can significantly reduce muscle fatigue without affecting work productivity. This will allow material handling workers to recover from muscle fatigue during work shifts and reduce the risk of WMSDs. These results confirmed the findings of other works [16,17,18,19,20,21] with objective measurement of muscle activity and body posture in the long term. We did not observe evidence of further effectiveness of the stretching routine during microbreaks. Our findings also demonstrated that the body posture characterization based on the K-score is an accurate, sensitive, and practical measurement tool for muscle fatigue during repetitive tasks. This is an important step in proving the efficacy of wearable sensors (IMU and EMG) in detecting muscle fatigue during material handling tasks and shows the potential of IMUs in substituting EMG sensors toward providing a reliable tool for long-term use in industrial environments.

## Figures and Tables

**Table 1:** Muscle fatigue measured with EMG amplitude and body posture (measured with K-score) in the three work–rest schedules for all participants.

	Fatigue Measured with EMG Amplitude (%)			Fatigue Measured with Body Posture (%)		
Participants	Schedule 1	Schedule 2	Schedule 3	Schedule 1	Schedule 2	Schedule 3
1	96.3	69.4	17.1	87.5	26.9	11.8
2	301.0	52.8	41.5	43.8	19.3	32.9
3	222.4	38.6	33.6	11.4	26.3	1.5
4	164.6	66.6	14.3	42.7	14.3	2.8
5	134.3	29.5	66.8	40.1	25.6	8.5
6	133.7	8.9	57.6	40.8	38.6	4.9
7	60.7	54.0	107.6	30.7	19.8	29.8
8	111.7	128.0	135.7	54.6	35.1	15.6
9	168.0	15.7	230.8	29.9	6.5	19.2
25th percentile	111.7	29.5	33.6	30.7	19.3	4.9
Median	134.3	52.8	57.6	40.8	25.6	11.8
75th percentile	168.0	66.6	107.6	43.8	26.9	19.2

**Table 2:** Number of repetitions (moving the box from one table to the other and back) made during each work–rest schedule, and the time until fatigue, are reported in Schedule 1.

Participants	Schedule 1	Schedule 2	Schedule 3	Time
1	198	234	245	30
2	146	162	187	30
3	97	93	108	15
4	232	293	192	30
5	235	209	166	35
6	417	279	441	30
7	309	345	266	40
8	545	981	768	60
9	384	371	455	50
25th percentile	198	209	187	30
Median	235	279	245	30
75th percentile	384	345	441	42.5

## Data Availability

The data presented in this study are available on request from the corresponding author. The data 231 are not publicly available due to privacy.
